# Phage therapy for extensively drug resistant *Acinetobacter baumannii* infection: case report and *in vivo* evaluation of the distribution of phage and the impact on gut microbiome

**DOI:** 10.3389/fmed.2024.1432703

**Published:** 2024-12-20

**Authors:** Jiayao Qu, Jin Zou, Jiancong Zhang, Jiuxin Qu, Hongzhou Lu

**Affiliations:** ^1^Department of Clinical Laboratory, National Clinical Research Center for Infectious Diseases, Shenzhen Third People’s Hospital, The Second Affiliated Hospital of Southern University of Science and Technology, Shenzhen, China; ^2^National Clinical Research Centre for Infectious Diseases, Shenzhen Third People’s Hospital and The Second Affiliated Hospital of Southern University of Science and Technology, Shenzhen, China

**Keywords:** inhaled phage therapy, extensively drug resistant (XDR), *Acinetobacter baumannii*, distribution, infection, gut microbiota

## Abstract

Numerous studies have documented successful instances of bacteriophage therapy in treating infections caused by extensively drug-resistant *Acinetobacter baumannii* (XDRAB). However, the safety profile of phage therapy and its effects on the human gut microbiota remain areas of concern. In this study, we collected blood, sputum, and fecal samples from an elderly female patient during two phases of inhaled bacteriophage therapy targeting extensively drug-resistant *Acinetobacter baumannii* (XDRAB). We investigated the *in vivo* distribution of bacteriophages and their impact on the gut microbiome. Bacteriophage DNA was detected in blood samples exclusively during the first 4 days of the second phase of phage therapy, with Ct values ranging from 32.6 to 35.3. In sputum samples, the Ct values of phages demonstrated a decreasing trend from 45 to 14.7 during the first phase of phage therapy, subsequently stabilizing between 28.5 and 26.8 in the second phase. In fecal samples, a significant reduction in the Ct value of phages was observed following both phases of bacteriophage treatment, with values decreasing from 35.5 to 22.5 and from 32.6 to 22.7, respectively. The composition of the gut microbiota was analyzed using Illumina-based 16S rRNA sequencing from fecal samples. Sequencing analysis revealed significant alterations in the microbiota composition at both the phylum and genus levels during phage therapy. These findings suggest that inhaled phages are detectable in human blood and tend to accumulate in the intestines. Furthermore, notable changes in the gut microbiota were observed throughout the duration of the phage treatment.

## 1 Introduction

The increasing prevalence of multidrug-resistant (MDR) bacterial strains, coupled with the sluggish advancement in the development of novel antibiotics, has rekindled significant interest in the application of phage therapy for the treatment of bacterial infections within clinical practice (1–3). Bacteriophages, or bacterial phages, are host-specific viruses that parasitize bacteria and replicate by utilizing the host’s metabolic pathways, ultimately resulting in the host’s death. These phages demonstrate a specificity for host infection that operates independently of bacterial antibiotic resistance mechanisms, enabling them to infect and lyse even antibiotic-resistant superbugs (4, 5).

Previous studies have demonstrated the potential efficacy of phage therapy in addressing infections caused by drug-resistant bacteria. For instance, the intravenous administration of phages into abscess cavities has been shown to reverse deteriorating conditions and inhibit or eradicate infections caused by multidrug-resistant *Acinetobacter baumannii* (MDRAB) (6). Additionally, another study documented the successful treatment of a patient infected with MDRAB through a combined approach involving antibiotics, intravenous phage injections, and aerosolized phage therapy (7). Several studies have documented the potential of phage therapy in addressing infections caused by multi-drug resistant bacteria (8–12). However, the extant literature predominantly emphasizes the clinical outcomes in patients, with relatively few investigations examining the biodistribution of phages within the host organism. For instance, in a study where phage therapy was administered to mice via the pulmonary route, phages were subsequently detected in the bloodstream (13). Similarly, another study involving the inhalation of phages by mice also reported the presence of phages in the serum (14). However, in a study investigating inhaled bacteriophage therapy using a porcine model of pneumonia, no infectious phages were detected in the serum (15). Furthermore, most clinical reports on inhaled phage therapy have not examined the distribution of phages within the bloodstream. Specifically, a study on bacteriophages reported an absence of bacteriophages in six blood samples (16). Although some studies have partially evaluated the distribution of phages, it remains uncertain whether inhaled phages have the potential to enter the bloodstream.

Different routes of administration lead to varying distributions of phages within tissues and distinct elimination processes. Bacteriophages that traverse the digestive tract initially encounter the acidic milieu of the stomach, presenting a significant barrier to their survival. Subsequently, within the intestines, the resident gut microbiota and the intestinal immune system actively surveil and eliminate phage-infected bacteria, thereby indirectly facilitating the clearance of the phages (17). Bacteriophages that enter the respiratory tract are initially subjected to the mucus-ciliary clearance mechanism, which facilitates the transport of foreign particles, including phages, towards the throat for expulsion. In the lower respiratory tract and lungs, immune cells such as macrophages play a crucial role in phagocytosing and eliminating infected bacteria, thereby restricting the dissemination of phages within the host organism (17). Additionally, the immune system is integral to the clearance of bacteriophages from the human body. Even in the absence of a specific immune response to phages, macrophages, monocytes, and phagocytes are actively involved in this process (18). Phagocytes also present phage antigens via antigen-presenting cells. The reticuloendothelial system (RES) of the spleen and liver serves as the primary immune mechanism responsible for phage clearance, effectively reducing phage concentrations to clinically useful levels (19). A study suggests that B cells are implicated in the clearance of bacteriophages, likely through phage-specific interactions (20). Additionally, there is evidence indicating that the kidneys also contribute to phage clearance, albeit to a lesser degree.

Numerous case reports have documented the application of phage therapy via inhalation; however, these studies have not examined the impact of inhaled phage therapy on the intestinal microbiota (21). In a study investigating bacteriophage therapy for *Escherichia coli* infection in rabbits, researchers observed that oral bacteriophage administration effectively treated intestinal infections while exerting minimal impact on the cecal microbiota (22). Experimental data indicated that oral phage administration altered the diversity of the gut microbiota (23). However, the overall impact of oral phage administration on gut bacteria remains inconclusive. Additionally, the effects of inhaled phage therapy on intestinal bacteria have yet to be determined. Further study is necessary to elucidate the effects of phage therapy on the human microbiota (24).

In this study, we reported a patient with a pulmonary infection caused by extensively drug-resistant *Acinetobacter baumannii* (XDRAB) who underwent phage therapy. Additionally, we assessed the presence of inhaled phages in the patient’s biological samples and examined the effects of phage therapy on the patient’s gut microbiota.

## 2 Methods

### 2.1 Phage screening and preparation

The XDRAB strain, isolated from a sputum sample, was utilized for phage screening at the Shenzhen Institute of Advanced Technology, Chinese Academy of Sciences (25). Phage BA3, which demonstrated significant specific lytic activity against XDRAB, was selected for further study. The phage preparation was subsequently purified using a cesium chloride density gradient, followed by dialysis with a Spectra/Por6 membrane in SM buffer (excluding Tris-HCl) to eliminate residual cesium chloride. The phages were then sterilized through filtration with 0.22 μm filters, and the purified phage preparation was stored at 4°C until required for subsequent applications.

### 2.2 Sample collection and DNA extraction

Sputum, plasma, and fecal samples were collected in sterile tubes in accordance with standard clinical procedures and subsequently stored at −80°C. DNA from sputum samples and cell-free DNA (cfDNA) from plasma samples were extracted using the PathoXtract^®^ Basic Pathogen Nucleic Acid Kit (WYXM03211S) and the PathoXtract^®^ Cell-Free Nucleic Acid Kit (WYXM03010S), respectively, following the manufacturer’s protocols. Total DNA from fecal samples was extracted using the E.Z.N.A.^®^ Soil DNA Kit (Omega Bio-tek, Norcross, GA, United States).

### 2.3 qPCR assay

*Acinetobacter baumannii* was quantified utilizing the *A. baumannii* Probes-Based Fluorescent Quantitative PCR Assay Kit (CS15-520) from Shanghai C-reagent Biotechnology Co., Ltd. The reaction mixture comprised 12.5 μL of 2× Universal Master Mix (Life Technology), 200 nM of both forward and reverse primers, 100 nM of probes, and 4 μL of template DNA, with water added to achieve a final reaction volume of 25 μL. The primers employed for the identification of *A. baumannii* phage were AB-pair1-F (5′-GCCATTCGACCATGCGTTAC-3′) and AB-pair1-R (5′-GTCGGATAAAAGCGAACCGC-3′). The reaction mixture comprised 12.5 μL of 2× SYBR GREEN UNIVERSAL MASTER MIX (4344463, Thermo Fisher), 200 nM of each primer, and 4 μL of template DNA. The volume was adjusted to a final reaction volume of 25 μL by adding water. qPCR was conducted using the Life Technologies ABI 7500 System platform. The thermal cycling conditions were as follows: initial activation of TaqMan at 95°C for 10 min, followed by 45 cycles of denaturation at 95°C for 10 s, and annealing/extension at 60°C for 60 s. The undetectable level of fluorescence was set at a cycle threshold (Ct) of 45.

### 2.4 Amplicon sequencing and data processing

The hypervariable V3–V4 region of the bacterial 16S rRNA gene was amplified using primer pairs 338F (5′-ACTCCTACGGGAGGCAGCAG-3′) and 806R (5′-GGACTACHVGGGTWTCTAAT-3′). The PCR reaction mixture comprised 4 μL of 5× Fast Pfu buffer, 2 μL of 2.5 mM dNTPs, 0.8 μL of each primer (5 μM), 0.4 μL of Fast Pfu polymerase, 10 ng of template DNA, and ddH_2_O to achieve a final volume of 20 μL. The PCR amplification cycling conditions were as follows: an initial denaturation at 95°C for 3 min, followed by 27 cycles consisting of denaturation at 95°C for 30 s, annealing at 55°C for 30 s, and extension at 72°C for 30 s. This was followed by a final extension at 72°C for 5 min, with the reaction terminating at 4°C. In the second round of PCR, index sequences were appended to the termini of the amplicons produced during the initial PCR using primers from the Nextera XT Index Kit (Illumina Inc., San Diego, CA, United States). The PCR amplification was conducted with the following reaction mixture: 10 μL of H_2_O, 20 μL of 5Prime Hot Master Mix, 5 μL of 1 μM forward primer, 5 μL of 1 μM reverse primer, and 10 μL of template pool at a concentration of 1 ng/μL. The amplification protocol employed was as follows: an initial denaturation at 94°C for 3 min; followed by 8 cycles of 94°C for 10 s, 58°C for 30 s, and 72°C for 45 s; and a final extension at 72°C for 10 min.

Purified amplicons were combined in equimolar concentrations and subjected to paired-end sequencing using the Illumina NovaSeq PE250 platform (Illumina, San Diego, United States). The resulting sequences were analyzed utilizing the plugin tools provided within the Quantitative Insights Into Microbial Ecology (QIIME2) bioinformatics package (version 2019.1). Two FASTQ files per sample (demultiplexed, paired-end reads) were imported into the QIIME2 environment. The DADA2 denoise-paired plugin was employed to: (i) trim primer sequences and low-quality bases at the read ends, (ii) join paired-end reads, (iii) discard chimeras, and (iv) infer amplicon sequence variants (ASVs). Additional chimera filtering was conducted using the VSEARCH uchime-denovo plugin. ASVs with fewer sequences than 0.005% of the total number of sequences and those not present in at least two samples were subsequently discarded. Taxonomic classification was performed utilizing the feature-classifier classify-sklearn plugin in conjunction with a Naïve Bayes classifier that had been pre-trained on the comprehensive Greengenes 13_8 99% OTU reference database (accessible at http://qiime2.org). ASVs identified as mitochondria, chloroplasts, or archaea were excluded, along with classifications that were resolved only to the level above phylum. Alpha and beta diversity metrics for each sample were calculated using QIIME with default parameters.

## 3 Results

### 3.1 General condition and clinical data

The patient, a 71-year-old female, has a medical history that includes a diagnosis of type 2 diabetes, lower limb venous thrombosis, and severe fatty liver disease. Subsequently, she developed weakness and numbness in the upper limbs, which gradually worsened, along with weakness in the left lower limb. She was diagnosed with ependymoma and underwent surgical treatment. Prior to this hospitalization, the patient experienced fever and cough for 15 days. She had been hospitalized three times for severe pneumonia caused by *Acinetobacter baumannii*, *Nocardia*, *Pneumocystis jirovecii*, and *Aspergillus*, and had received long-term combination antibiotic treatments. Following hospitalization, she was placed on mechanical ventilation and administered intravenous voriconazole at a dosage of 150 mg every 12 h. A bronchoalveolar lavage fluid (BALF) culture, collected 4 days prior to the initiation of the first phase of phage therapy, tested positive for XDRAB. Following the signing of the informed consent form by the legal representative, the patient received two courses of phage therapy in conjunction with antibiotics. The first phase of phage therapy involved the administration of 0.5 mL × 10^9^ PFU/mL via inhalation, twice daily, from January 8, 2022, to January 17, 2022 (a total duration of 10 days). However, 12 days after the completion of the first course of phage therapy, an increased burden of *Acinetobacter baumannii* was detected in the BALF culture, and *Pseudomonas aeruginosa* was also cultured. Nevertheless, based on the clinical condition, clinicians did not make further adjustments to the patient’s antibiotic regimen. Then, the patient commenced the second course of phage therapy on January 29, 2022, which continued until February 7, 2022, spanning a total of 10 days. During this phase, the treatment protocol for the initial 3 days entailed the administration of 0.3 mL of 10^10^ PFU/mL BA3 phage, diluted in 4.7 mL of saline, delivered via mechanical ventilation twice daily. From the fourth day onward, the regimen was adjusted to administer 0.1 mL of 10^10^ PFU/mL phage, diluted in 4.9 mL of saline, via inhalation twice daily. During the two courses of phage therapy, we recorded changes in the patient’s temperature, total white blood cell count, neutrophil percentage, C-reactive protein (CRP), procalcitonin (PCT), and interleukin-6 (IL-6) (Supplementary Figure S1). Throughout both rounds of phage therapy, the patient’s body temperature remained within normal limits (<37.3°C). Aside from an elevation in the WBC count observed on the seventh day of the first round, the WBC count remained within the normal range. The percentage of neutrophils consistently exceeded 75% during both rounds of therapy. Inflammatory markers, including CRP, PCT, and IL-6, exhibited an increase on the seventh and eighth days of the first round. During the second round of therapy, these inflammatory markers initially increased but subsequently declined (Supplementary Figure S1). Despite the fluctuations in inflammatory markers observed during the second round of phage therapy, clinicians evaluated the patient’s condition as stable and opted not to implement additional interventions. No severe adverse reactions were noted throughout the course of treatment.

### 3.2 Bacteriophage detection in human samples

**Figure 1:**
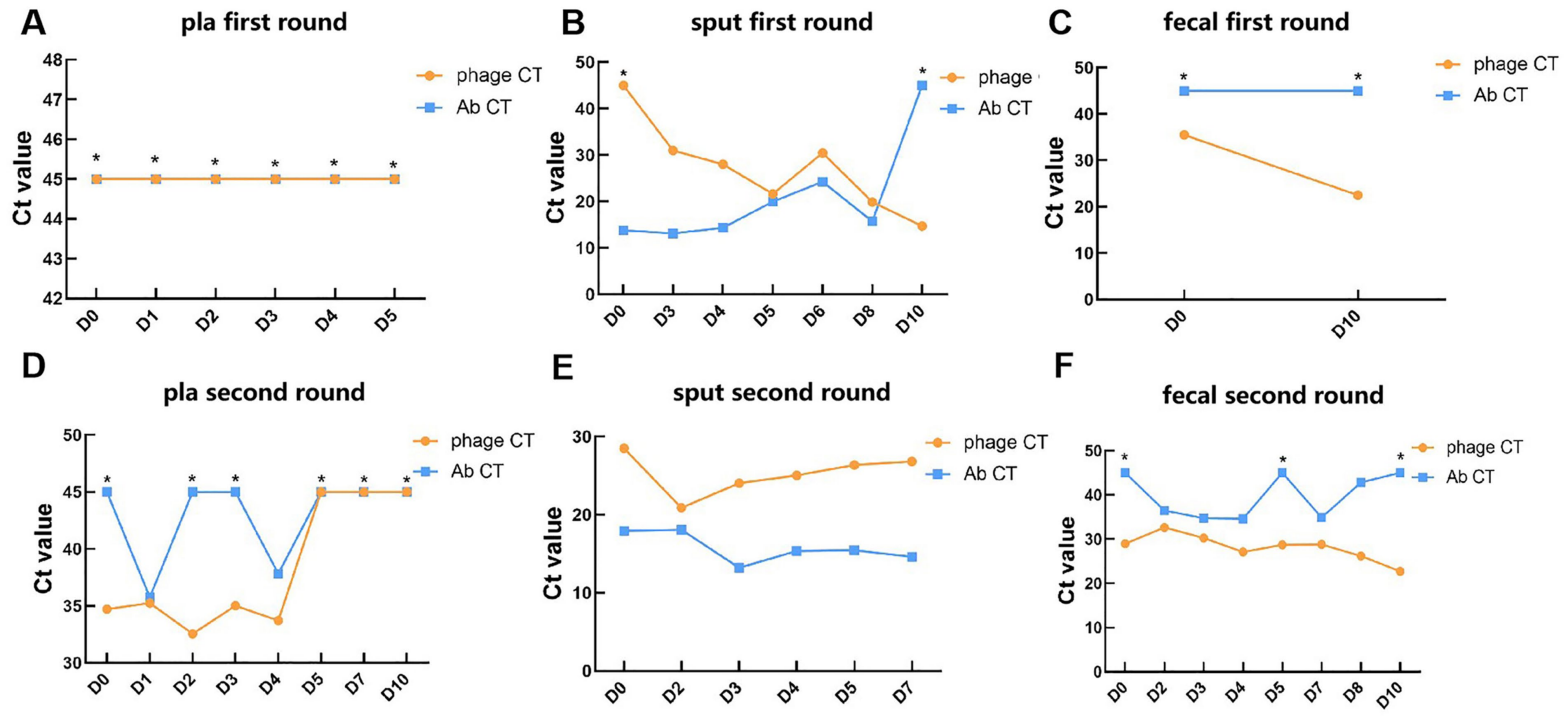
Detection of phages and targeted pathogen in clinical samples during phage therapy. **(A–C)** The Ct values of phages and *A. baumannii* in plasma, sputum, and fecal samples of patients in the first round of phage therapy. **(D–F)** The Ct values of phages and *A. baumannii* in plasma, sputum, and fecal samples of patients in the second round of phage therapy. *: Negative results determined as 45. Key: Ct value <20: very high relative abundance. Ct value 20–26: high relative abundance. Ct value 26–32: moderate to high relative abundance. Ct value 32–38: low relative abundance. Ct value 38–45: very low relative abundance. Ct value ≥45: undetectable relative abundance.

Phages and pathogen DNA were initially identified in 14 blood samples, 13 sputum samples, and 10 fecal samples collected across two phases of phage treatment using real-time PCR. The relative abundance of the detected pathogens or phages, contingent upon the specific target, is represented by the Ct value. In the blood samples, negative PCR results were predominantly observed for *A. baumannii* and the bacteriophage DNA, with exceptions noted for the pathogen on day 1 (Ct = 35.8) and day 4 (Ct = 37.8) during the second phase, and for bacteriophages (Ct range: 32.6 to 35.3) within the first 4 days of the second phase (Figures 1A,D). In the sputum samples, there was a gradual increase in the abundance of bacteriophages in the patient’s respiratory tract, as indicated by a decreasing trend in Ct values from 45 to 14.7 during the first phase (Figure 1B). Concurrently, the Ct value of *A. baumannii* exhibited a gradual increase from 13.8 on day 0 to 45 on day 10, indicating a negative result (Figure 1B). Unexpectedly, during the second phase, the Ct values of the phage remained between 28.5 and 26.8, while the Ct values of the pathogen, *A. baumannii*, were relatively lower, ranging from 13.2 to 18.1 (Figure 1E). This observation suggests that the phages did not exhibit significant lytic activity against the pathogen in the second phase, thereby highlighting the critical issue of phage resistance in the context of phage therapy (26). In the fecal samples, the Ct value of the phage on the 10th day post-treatment (22.5) was lower than that prior to the initial treatment (35.5) (Figure 1C). This value stabilized at 29 before the commencement of the second phage treatment, subsequently decreasing during the second phase of phage administration (from 32.6 to 22.7) (Figure 1F), indicating an increased phage presence in the patient’s gut. Regarding the pathogen, its DNA was detected in only 5 samples from the second phase, exhibiting relatively higher Ct values (34.6–36.5) (Figures 1C,F). This finding suggested that bacteriophages through inhalation may enter and accumulate in the human intestine.

### 3.3 Changes in gut microbiota during phage therapy

**Figure 2:**
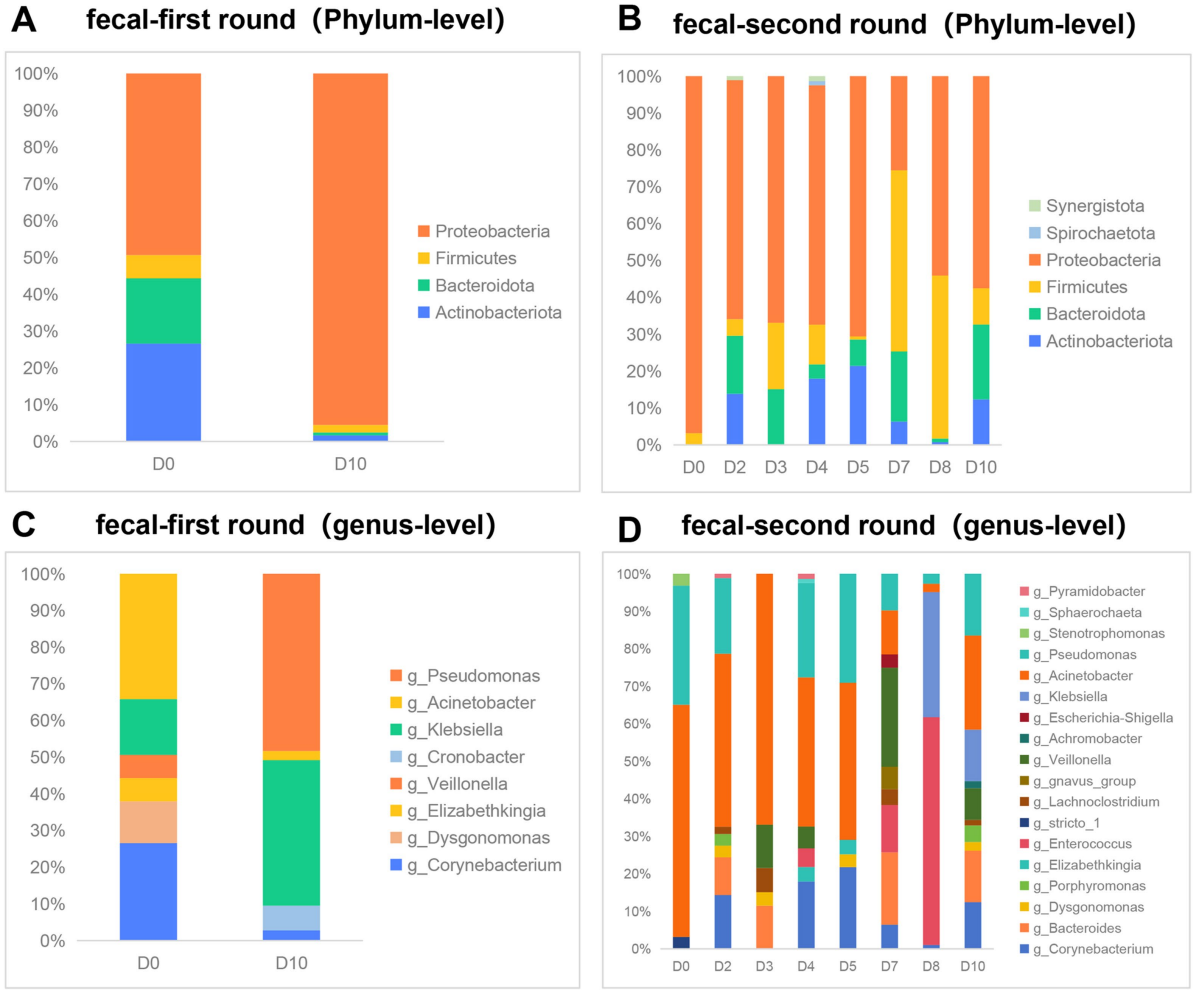
Effect of phage therapy on gut microbiota composition in patient. **(A,B)** Microbial community bar plot with the relative abundance higher than 0.05% in fecal samples at the phylum level in the first round of phage therapy and in the second phase. **(C,D)** Microbial community bar plot at the genus level with the relative abundance higher than 1% in the first round of phage therapy and in the second phase.

We conducted an analysis of the gut microbiota composition in 10 fecal samples utilizing Illumina-based 16S rRNA sequencing. Our observations indicated that, at a relative abundance threshold of ≥0.05% at the phylum level, there was a substantial increase in the proportion of *Proteobacteria* (from 49.4 to 95.5%). Concurrently, the relative abundances of the other three dominant phyla—*Actinobacteriota*, *Bacteroidota*, and *Firmicutes*—exhibited varying degrees of reduction during the first phase of phage therapy (Figure 2A). Similar to the first phase, *Proteobacteria* maintained a dominant position; however, the relative abundance of the four predominant phyla—*Proteobacteria*, *Actinobacteriota*, *Bacteroidota*, and *Firmicutes*—exhibited fluctuations during the second phase of phage application (Figure 2B). At the genus level, with a relative abundance of ≥1%, the predominant genera prior to the first round of phage therapy were *Acinetobacter* (34%), *Corynebacterium* (27%), and *Klebsiella* (15%). Following treatment, these shifted to *Pseudomonas* (48%) and *Klebsiella* (40%) (Figure 2C). Prior to the second phase of phage treatment, *Acinetobacter* and *Pseudomonas* constituted 61.9 and 31.7% of the microbial community, respectively. Notably, the *genera stricto* and *Stenotrophomonas* exhibited a reduction in abundance to undetectable levels. Conversely, 13 other genera demonstrated an increase in abundance following phage therapy. These genera include *Corynebacterium*, *Bacteroides*, *Dysgonomonas*, *Porphyromonas*, *Elizabethkingia*, *Lachnoclostridium*, *gnavus*, *Veillonella*, *Achromobacter*, *Escherichia*, *Klebsiella*, *Sphaerochaeta*, *Pyramidobacter*, and *Enterococcus* (Figure 2D).

## 4 Discussion

Following two rounds of phage therapy and continuous clinical antibiotic treatment as part of a comprehensive therapeutic regimen, the patient’s condition stabilized compared to her initial admission. Before discharge, the patient was in a post-tracheotomy state and had successfully been weaned off the ventilator. The patient exhibited no symptoms of fever, abdominal pain, chest tightness, or chest pain, and her mental state and sleep quality were reported to be satisfactory. With medical authorization, the patient was transferred to a rehabilitation hospital for ongoing care. Prior to the patient’s second round of phage therapy, *Pseudomonas aeruginosa* was also detected. Based on the patient’s condition, clinicians did not adjust the antibiotic treatment regimen. During the second round of phage therapy, the patient’s infection markers (CRP, IL-6) initially increased and then decreased, which may be related to the immune system’s response to *Pseudomonas aeruginosa*.

In this study, we were unable to detect phages in blood samples during the first round of phage treatment. However, trace amounts of bacteriophage DNA sequences were identified during the second phase of phage therapy. Notably, previous research has detected the presence of bacteriophages in blood samples (27, 28). Nevertheless, the question of whether inhaled phages can enter the bloodstream remains a topic of ongoing debate. In alignment with this finding, Chang et al. (13) identified the presence of phages in the blood of mice following pulmonary administration of phages for the treatment of *Pseudomonas aeruginosa* pulmonary infections. Similarly, another investigation into phage therapy within a murine model of *Pseudomonas aeruginosa* infection also documented the presence of bacteriophages in the serum (14). Conversely, a study examining inhaled bacteriophage therapy in a porcine model of pneumonia did not detect any infectious phages in the serum (15). Our findings indicate that prolonged inhalation of the phage may facilitate its dissemination into the bloodstream; however, additional clinical trials are necessary to thoroughly assess the safety and implications of this phenomenon. Furthermore, our data demonstrated a progressive decline in phage Ct values in the gut following phage therapy, suggesting an accumulation of phages within the gastrointestinal tract. The mechanisms underlying the distribution of inhaled bacteriophages in the gut, however, remain to be elucidated. Upon inhalation into the lungs, bacteriophages may be translocated to the gastrointestinal tract through internal cellular mechanisms (18), or alternatively, some inhaled bacteriophages may access the upper digestive tract via the oral cavity, ultimately reaching the intestines (17). The interactions between bacteriophages and the gut microbiota are notably intricate. The presence of active bacteriophages in the gut may influence the equilibrium of intestinal bacterial populations (29). Given the limited sample size, we have only performed qPCR detection on bacteriophage DNA in fecal samples. Consequently, we are unable to confirm the presence of viable bacteriophages in the intestine. Assuming the presence of live bacteriophages in the intestinal environment, further research is necessary to elucidate the mechanisms by which inhaled bacteriophages traverse to the intestine. Additionally, it is imperative to assess the safety of bacteriophage inhalation therapy and its potential effects on intestinal microecology.

The composition of a healthy gut microbiota predominantly includes bacteria from four phyla: *Firmicutes* (60 to 65%), *Bacteroidetes* (20 to 25%), *Proteobacteria* (5 to 10%), and *Actinobacteria* (3%) (30). However, it is important to consider that the patient’s extended antibiotic treatment may have altered her gut microbiota composition prior to the administration of phage therapy, potentially deviating from the typical profile observed in healthy individuals. Our findings indicate that the predominant bacterial phyla identified were *Actinobacteria*, *Bacteroidetes*, *Firmicutes*, and *Proteobacteria*, aligning with the composition of a healthy gut microbiota (30). However, in contrast to a healthy gut microbiota, the patient’s intestines exhibited a marked predominance of the *Proteobacteria* phylum, ranging from 49.4 to 95.5%. At the genus level, *Clostridium* constitutes 95% of the phylum *Firmicutes* within the normal intestinal microbiota, with *Bacteroidetes* being the subsequent predominant group. In contrast, the patient’s gut microbiota was primarily composed of the genera *Acinetobacter*, *Pseudomonas*, and *Klebsiella*. Notably, the relative abundance of these genera exhibited significant perturbations during the two phases of phage therapy. The influence of bacteriophages on the composition of intestinal microbiota remains a subject of ongoing debate. A study investigating bacteriophage therapy for *Staphylococcus aureus* device infections reported no significant differences in bacterial abundance within the fecal samples of the patients (31). In a murine experimental study, researchers observed an increase in the richness and diversity of the microbial flora in the feces of mice treated with phages (23). Additionally, Febvre et al. (32) reported that while phage treatment did not induce global changes in the microbiota, it did result in significant alterations in specific microbial community members. Notably, the abundance of *E. coli*, the target host for the administered phage consortium, was markedly reduced by the conclusion of the treatment period.

Prolonged antibiotic administration significantly impacts the composition of the gut microbiota (33). The patient, a chronic lung disease sufferer, had been subjected to extended antibiotic treatment prior to admission, which included voriconazole, imipenem, tigecycline, and piperacillin-tazobactam. Antibiotic therapy was maintained post-admission (Supplementary Figure S2). Consequently, it is anticipated that the gut microbiota experienced disruption due to antibiotic exposure. In this study, a comparative analysis of the gut microbiota before and after phage therapy revealed a reduction in the proportion of the genus *Acinetobacter* following treatment. Previous research has demonstrated that viruses are capable of translocating across mucosal barriers to reach distal tissues (34). Inhaled bacteriophages may traverse specific pathways to enter the gastrointestinal tract, potentially altering the composition of the gut microbiota. However, it is important to note that the patient was concurrently administered multiple antibiotics during the course of phage therapy, with adjustments made to the antibiotic regimen based on the patient’s clinical condition. Consequently, the impact of antibiotics on the changes in the patient’s gut microbiota is challenging to quantify or exclude. Our findings, as indicated by Ct values, suggest the accumulation of inhaled phages in the gastrointestinal tract. However, the detection of phage DNA in fecal samples via qPCR does not confirm the presence of viable phages within the gut. Our study was conducted with a single patient, presenting a limited sample size. Consequently, it is challenging to attribute the observed alterations in gut microbiota to the administration of phages based on this isolated case. Further research is warranted to elucidate the potential effects of inhaled bacteriophages on gut microbiota.

Bacteria can acquire resistance to bacteriophages through multiple mechanisms, including the superinfection exclusion system (Sie) and the CRISPR-Cas system, among others (35, 36). Previous study has demonstrated that extended phage therapy can result in the development of phage tolerance (26). During the first phase of phage therapy in this study, the Ct values of the target bacteria in the patient’s sputum exhibited an upward trend as the treatment advanced. Conversely, the Ct values of the phages demonstrated a decline, indicating successful proliferation of the phages within the bacterial population. This observation suggests a certain degree of efficacy of phage therapy during the first phase of phage treatment. However, during the second round of phage therapy, the Ct values of the target bacteria in the patient’s sputum remained relatively stable and low. Concurrently, the phage Ct values also remained stable but at relatively high levels (28.5–26.8) compared to the first phase. This suggests that the phages did not proliferate effectively within the pathogens during the second phase of treatment. Additionally, *Pseudomonas aeruginosa* was detected in the patient prior to the second round of phage therapy, which may be related to the patient’s longer hospital stays. Based on these results, we speculate that the extended administration of phages may have contributed to the development of phage tolerance in the patient. Consistent with our findings, Bao et al. (37) observed the emergence of phage-resistant mutants within a few days during two rounds of phage therapy, highlighting the critical issue of phage resistance in such treatments. Additionally, multiple studies have documented instances of phage therapy where phage-resistant variants were identified (6, 38, 39).

In conclusion, our results suggest the presence of inhaled bacteriophages in human blood and their subsequent accumulation in the intestines. Notably, we observed a stabilization in phage abundance and a sustained high burden of pathogens following the first course of phage treatment. This observation suggests that prolonged phage therapy may induce phage resistance, thereby diminishing its therapeutic efficacy. Our case study also delineates the alterations in relative abundance and diversity at the genus level of the gut microbiome during phage therapy. While numerous studies have indicated that phage therapy may be safe in clinical practice, further clinical trials are necessary to assess the safety and implications of these therapeutic strategies.

## Data Availability

The raw data supporting the conclusions of this article will be made available by the authors, without undue reservation.

## Supplementary material

SUPPLEMENTARY FIGURE S1 Clinical indicators of the patient during bacteriophage therapy. **(A,B)** WBC counts, body temperature and antibiotics usage during the period of first and second round phage treatment, respectively. **(C,D)** The changes of the percentage of neutrophils, CRP, IL-6, and PCT during the period of first and second round phage treatment, respectively.

SUPPLEMENTARY FIGURE S2 The antibiotic usage during the patient's hospitalization and the sample collection during phage therapy. LZD, linezolid; PMB, polymyxin B; TGC, tigecycline; SULPERAZON, cefoperazone sodium and sulbactam sodium for injection; TZP, tazocin; CAZ-AVI, ceftazidime-avibactam. ◆: Sample collection. *D0: Samples were collected one hour before the patient received inhaled phage therapy. *D1: Samples were collected two hours after the patient received inhaled phage therapy.
