# Supplementary figures and images for: Phage therapy for extensively drug resistant Acinetobacter baumannii infection: case report and in vivo evaluation of the distribution of phage and the impact on gut microbiome
Source: Front Med (Lausanne). 2024 Dec 20;11:1432703. doi: 10.3389/fmed.2024.1432703 (PMC11695418; doi:10.3389/fmed.2024.1432703)

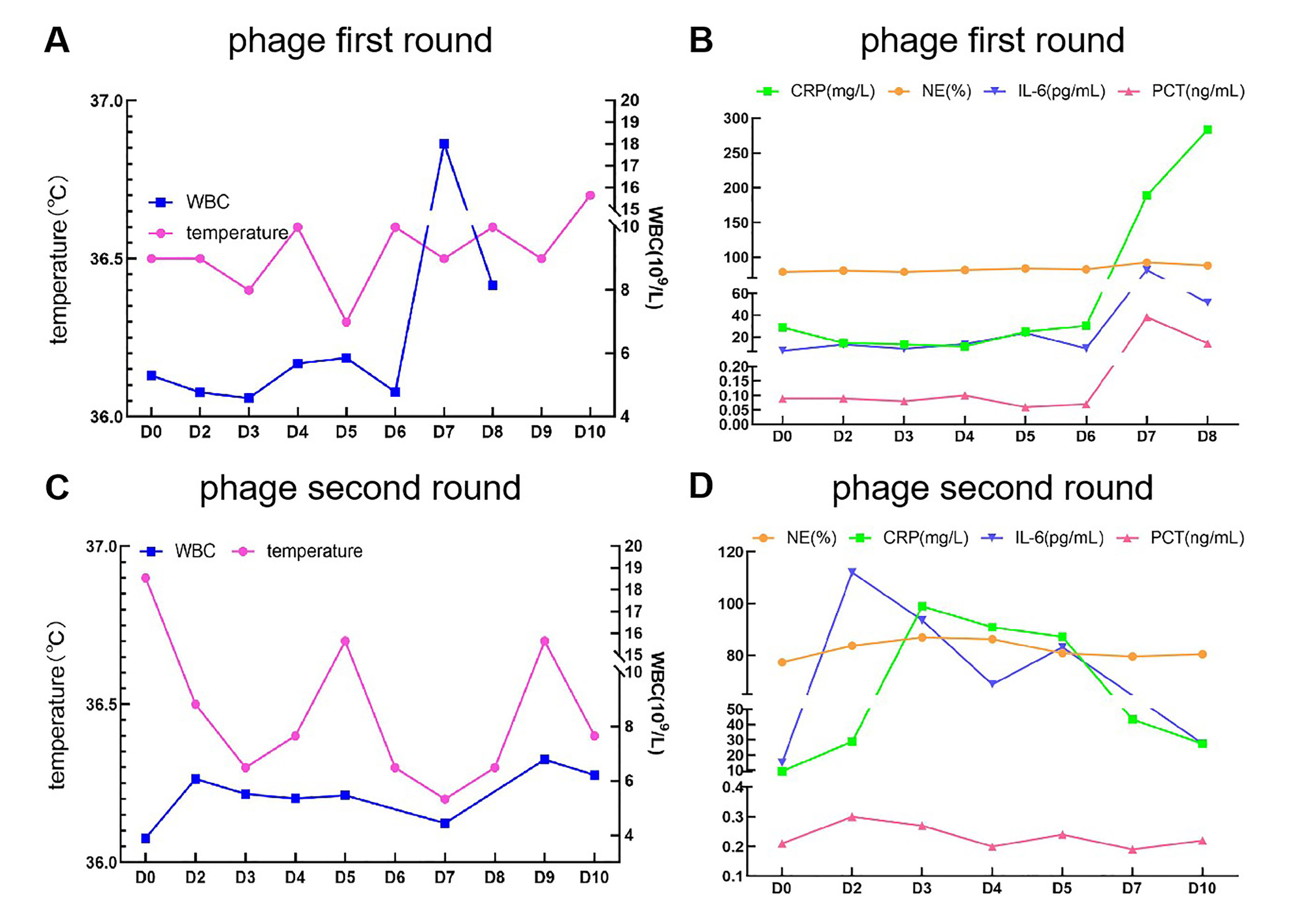

Supplement: SUPPLEMENTARY FIGURE S1 — Clinical indicators of the patient during bacteriophage therapy. (A,B) WBC counts, body temperature and antibiotics usage during the period of first and second round phage treatment, respectively. (C,D) The changes of the percentage of neutrophils, CRP, IL-6, and PCT during the period of first and second round phage treatment, respectively. [file Image_1.JPEG]

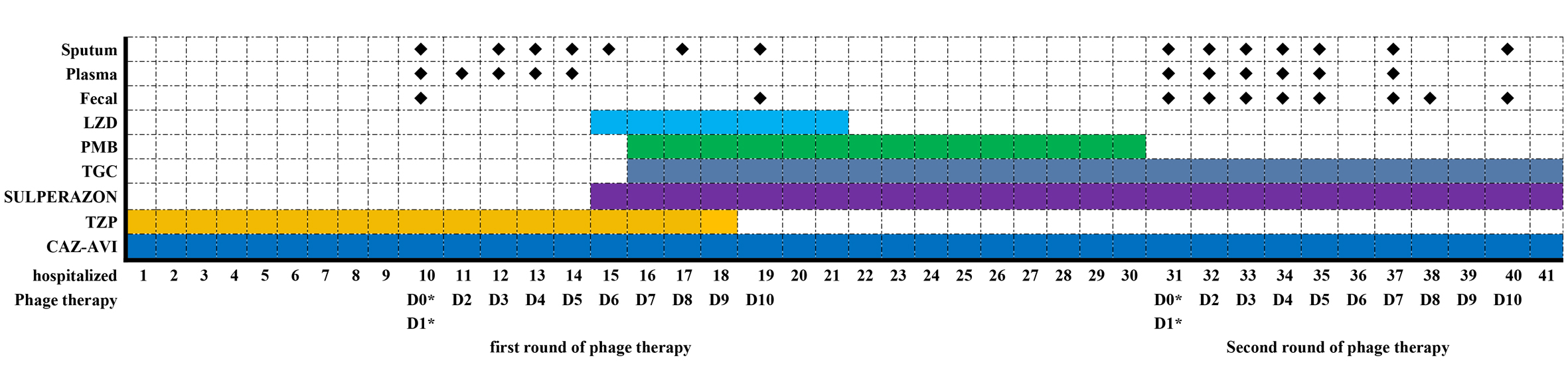

Supplement: SUPPLEMENTARY FIGURE S2 — The antibiotic usage during the patient's hospitalization and the sample collection during phage therapy. LZD, linezolid; PMB, polymyxin B; TGC, tigecycline; SULPERAZON, cefoperazone sodium and sulbactam sodium for injection; TZP, tazocin; CAZ-AVI, ceftazidime-avibactam. ◆: Sample collection. *D0: Samples were collected one hour before the patient received inhaled phage therapy. *D1: Samples were collected two hours after the patient received inhaled phage therapy. [file Image_2.JPEG]
